# Influence of Surface Modification of Fly Ashes on the Fire Behavior of Polyamide 6

**DOI:** 10.3390/polym18080970

**Published:** 2026-04-16

**Authors:** Marcos Batistella, Nour-Alhoda Masarra, Constantinos Xenopoulos, José-Marie Lopez-Cuesta

**Affiliations:** 1 Polymers Composites and Hybrids, IMT Mines Alès, 30319 Ales, France; nour.masarra2@gmail.com (N.-A.M.);; 2 Holcim Innovation Center, 38090 Saint Quentin Fallavier, France; constantinos.xenopoulos@holcim.com

**Keywords:** fly ash, polyamide 6, surface modification, fire behavior

## Abstract

This study investigates the influence of surface-modified fly ash particles on the fire behavior of polyamide 6 (PA6) composites containing two types of flame retardants: melamine polyphosphate (MPP) and aluminum diethyl phosphinate (AlPi). The objective was to evaluate how interfacial modification of fly ash using amino-silane (APTES), glycidoxy-silane (GPTES), or titanate coupling agents affects dispersion, thermal stability, and combustion performance. A series of 18 formulations containing up to 25 wt% of additives was prepared by melt compounding and characterized by thermogravimetric analysis (TGA) and cone calorimetry. TGA results showed that MPP-based systems favored char formation, with residues up to 21%, whereas AlPi provided higher thermal stability (T_50_% ≈ 445 °C). The incorporation of untreated or surface-treated fly ash improved both thermal stability and char yield, depending on the nature of the coupling agent. Cone calorimeter results confirmed a strong synergistic effect between flame retardants and fly ash. The peak heat release rate (pHRR) decreased by 65–75% compared to neat PA6, while total heat release (THR) and mass loss were also significantly reduced. Titanate-modified fly ash showed the most homogeneous dispersion and provided the highest residue and lowest pHRR values. Energy-dispersive X-ray (EDX) analyses confirmed enhanced phosphorus retention in the residues (up to 100%), evidencing the formation of stable inorganic species and protective ceramic-like structures. These results demonstrate that surface-modified fly ash can act as an efficient synergistic additive in PA6 flame-retardant formulations, simultaneously improving fire performance and promoting the valorization of industrial by-products for sustainable polymer design.

## 1. Introduction

Polyamides are used in various applications as engineering thermoplastics due to their excellent mechanical strength, thermal stability, and chemical resistance. However, their inherent flammability and dripping behavior under fire conditions limit their use in applications requiring high fire safety standards. To improve their flame retardancy, various strategies have been explored, including the incorporation of flame-retardant additives or the development of polymer composites with inorganic fillers. Various works in the literature evaluated the use of various flame retardants alone [1,2,3] or in combination with a multitude of fillers, especially organo-modified clays [4,5,6,7].

Various non-halogenated systems were proposed to improve the fire reaction of PA6, such as phosphorous (ammonium polyphosphates [2], red phosphorus [8], and metallic phosphinates [3,9,10]), nitrogenous compounds (melamine cyanurate [11,12] and melamine pyrophosphate [13,14]) as well as mineral fillers such as kaolinite [9,15] and montmorillonite [16].

In parallel with the growing interest in halogen-free flame-retardant polymers, recent studies increasingly emphasize the development of more sustainable formulations based on industrial by-products and waste-derived mineral fillers. Recent work on recycled ABS filled with fly ash microspheres showed that such waste-derived fillers can contribute to the development of value-added polymer composites with improved thermal stability while supporting waste valorization strategies [17]. In addition, Liu et al. [18] recently reported that fly ash cenospheres can be incorporated into multifunctional PLA composites with improved flame retardancy and thermal insulation, further illustrating the potential of this industrial residue in fire-safe polymer design. Surface modification also appears to be a key parameter: Wu et al. [19] showed that KH-550 functionalization of fly ash improved its compatibility and dispersibility in a polymer coating, resulting in a significant reduction in peak heat release rate. More broadly, the recent literature on inorganic flame-retardant synergists highlights that the efficiency of mineral additives strongly depends on their dispersion, interfacial interactions, and ability to promote protective barrier formation and smoke suppression during combustion. These recent advances therefore support the relevance of investigating fly ash as a sustainable synergistic component in flame-retardant PA6 and, more specifically, the role of fly ash surface treatment in controlling its effectiveness in phosphorus-based flame-retardant systems.

Several studies have investigated the incorporation of untreated or surface-modified fly ash into thermoplastic matrices, reporting improvements in mechanical properties [20,21,22,23,24,25] and, to a lesser extent, flame retardancy [26,27,28,29,30]. Nevertheless, research on the use of fly ash in polyamide-based systems remains limited, particularly regarding how surface modification influences the fire behavior of such composites. Beyond their technical potential, fly ash particles offer an attractive route for sustainable material design, as they are abundant industrial byproducts. Their use as fillers promotes the valorization of waste materials, reduces dependency on virgin resources, and supports the development of environmentally friendly polymer composites.

Therefore, this work aims to investigate the influence of fly ash surface modification on the fire behavior of PA6 composites containing either melamine polyphosphate or aluminum diethyl phosphinate. Untreated and surface-treated fly ashes, modified with amino-silane, glycidoxy-silane, or titanate coupling agents, were compared in order to evaluate the role of surface chemistry on filler dispersion and flame-retardant behavior. The originality of the study lies in the comparative analysis of these three surface treatments in two phosphorus-based flame-retardant PA6 systems, with particular emphasis on thermal degradation, cone calorimeter results, and phosphorus retention in the combustion residue.

## 2. Materials and Methods

### 2.1. Materials

The polyamide 6 (PA6) used in this study was a commercial grade Technyl^®^ C206, supplied by Rhodia (Lyon, France), with a density of 1.14 g/cm^3^. Several flame-retardant additives and surface modifiers were employed to investigate their effects on the fire performance of PA6. The melamine polyphosphate used in this study is referred to as MP200 (Melapur® 200 form BASF, Ludwigshafen, Germany). The aluminum diethylphosphinate-based flame retardant employed is a commercial product known as Exolit^®^ OP1230, supplied by Clariant (Munich, Germany). The mineral additive referred to as SuperPozz is a highly reactive pozzolanic material, composed mainly of amorphous aluminosilicates, selected for its potential to promote char formation and act as a physical barrier. SuperPozz, with a median particle diameter of 6 μm and a specific surface area of 1.55 m^2^/g, was supplied by Holcim (Saint Quentin Fallavier, France). Its detailed composition, determined by X-ray fluorescence and X-ray diffraction coupled with Rietveld analysis, has been reported in our previous work [28,29].

To enhance the compatibility of this filler with the polyamide matrix, various surface treatments were applied. 3-Glycidoxypropyltrimethoxysilane (GPTES) and 3-Aminopropyltrimethoxysilane (APTES) (≥98%, Sigma-Aldrich, St. Louis, MO, USA) and a titanate coupling agent, isopropyl tri(dioctylpyrophosphato)titanate (KR-12), were used as received.

All materials were used as received without further purification. Prior to compounding, PA6 pellets and fillers were dried under vacuum at 80 °C for at least 8 h to remove moisture and prevent hydrolysis during processing. A total of 18 formulations (PA6_a to PA6_s) were prepared to evaluate the influence of both the flame retardant type and the nature of the mineral filler—treated or untreated—on the fire behavior of PA6.

### 2.2. Surface Modification of Fly Ash

The surface of the fly ash particles was modified using three different coupling agents. The modification procedure was as follows: an ethanol/water solution (96:4 by volume) was first prepared, and its pH was adjusted to 4 using glacial acetic acid. Fly ash was then added at a concentration of 10 wt.% relative to the total solution mass. The suspension was heated to 80 °C under constant stirring. Once the temperature was stabilized, the selected surface modification agent was added at 1 wt.% with respect to the mass of fly ash. The mixture was maintained at 80 °C for 6 h to allow sufficient interaction between the particles and the coupling agent.

After the reaction, the modified fly ash was filtered and thoroughly washed with ethanol to remove any unreacted coupling agent. The powder was then dried in an oven at 100 °C for 8 h to eliminate residual solvents.

Taking into account the specific surface area of SuperPozz (1.55 m^2^/g) and the addition of 1 wt.% coupling agent relative to the fly ash mass, the nominal surface loading is approximately 6.45 mg/m^2^, corresponding to about 36 μmol/m^2^ for APTES, 27 μmol/m^2^ for GPTES, and 4.9 μmol/m^2^ for KR-12. This amount was considered sufficient to ensure complete surface coverage of the fly ash particles, whereas any unreacted or weakly adsorbed excess was assumed to be removed during the washing step.

### 2.3. Preparation of PA6 Composites

The formulations were prepared by melt compounding using a co-rotating twin-screw extruder (Clextral BC 21, Firminy-France) with a barrel length of 900 mm, a screw diameter of 25 mm, and an L/D ratio of 48. The extrusion was carried out at a screw speed of 250 rpm and a total throughput of 4 kg/h. The extruder was equipped with nine heating zones. The temperature profile was set to 60 °C in zone 1, 170 °C in zone 2, and 260 °C in zones 3 to 9. A vacuum pump was connected at the last zone of the extruder in order to limit the hot hydrolysis of PA6 during processing. After extrusion, all formulations were injection molded into 100 × 100 × 4 mm^3^ plates for cone calorimeter tests using a Krauss Maffei 50 t injection molding machine (Vaterstetten, Germany). The injection temperature was set at 260 °C and the mold temperature at 60 °C.

A series of polyamide 6 (PA6) formulations were prepared by incorporating a total of 25 wt% of additives, composed of flame retardants (FR) and fly ash (Super Pozz, abbreviated as P), either untreated or surface-modified. Two flame retardants were used: melamine polyphosphate (MP) and aluminum diethyl phosphinate (OP). The fly ash was used in its unmodified form (P) or treated with amino-silane (P-A), glycidoxy-silane (P-G), or titanate (P-T). Each formulation is denoted using a combination of abbreviations followed by their respective weight percentages.

Formulations containing only flame retardant were denoted as MP25 or OP25, depending on the type. Binary formulations, consisting of one flame retardant and untreated fly ash, include MP18.75-P6.25 and MP12.5-P12.5 for melamine polyphosphate, and OP18.75-P6.25 and OP12.5-P12.5 for aluminum phosphinate.

Formulations incorporating surface-treated fly ash are designated accordingly. For MPP systems:

- MP18.75-P-A6.25 and MP12.5-P-A12.5 include amino-silane-modified fly ash;
- MP18.75-P-G6.25 and MP12.5-P-G12.5 contain glycidoxy-silane-treated fly ash;
- MP18.75-P-T6.25 and MP12.5-P-T12.5 correspond to titanate-treated fly ash.

Similarly, OP-based formulations include:

- OP18.75-P-A6.25 and OP12.5-P-A12.5;
- OP18.75-P-G6.25 and OP12.5-P-G12.5;
- OP18.75-P-T6.25 and OP12.5-P-T12.5.

### 2.4. Characterization Methods

Thermogravimetric analysis (TGA) was performed using a thermal analyzer under a nitrogen atmosphere to evaluate the thermal stability of the samples. Approximately 10 mg of each sample was heated from 50 °C to 750 °C at a constant heating rate of 10 °C/min under a nitrogen flow of 40 L/min.

The fire behavior of the composites was assessed using two standard methods. First, cone calorimetry tests were conducted according to ISO 5660-1 [31] under an external heat flux of 50 kW/m^2^. Samples were exposed horizontally, and key parameters such as time to ignition (TTI), peak heat release rate (pHRR), total heat release (THR), and mass loss were recorded. UL-94 vertical burning tests (UL 94 [32]) were performed on 90 × 10 × 4 mm^3^ specimens to evaluate the self-extinguishing behavior of the materials. The highest classification, V-0, is obtained when the specimen extinguishes rapidly, exhibits only slight afterglow, and does not generate flaming drips. A V-2 rating corresponds to poorer performance, generally associated with longer burning times and the occurrence of flaming drips.

Scanning electron microscopy (SEM) observations were carried out with a Quanta 200 FEG microscope (FEI Company, Hillsboro, OR, USA) operated under high vacuum at an accelerating voltage of 12.5 kV and using BSE mode. SEM was used to examine both the dispersion of fillers in the polymer matrix and the morphology of the residues after cone calorimeter testing. Elemental analysis of the combustion residues was additionally performed by energy-dispersive X-ray spectroscopy (EDS) using an Oxford X-MaxN system (Oxford Instruments, Abingdon, UK) fitted with a detector of 133 eV resolution.

X-ray diffraction (XRD) was performed on the cone calorimeter residues in order to examine possible interactions between the flame retardants and fly ash after combustion. The analyses were conducted on a D8 Advance diffractometer (Bruker AXS, Billerica, MA, USA) equipped with CuKα radiation and a LynxEye detector, in the 5–70° 2θ range, using a step size of 0.007°.

## 3. Results

This section presents the evaluation of thermal and fire behavior of the different PA6-based formulations incorporating flame retardants and mineral fillers. The objective was to investigate the influence of both the flame retardant type (melamine polyphosphate or aluminum phosphinate) and the presence of untreated or surface-modified fly ash on the decomposition profile, char formation, and fire performance of PA6. Thermogravimetric analysis (TGA) was first used to characterize the thermal degradation behavior under an inert atmosphere. Subsequently, cone calorimetry tests were performed to assess the combustion properties under forced-flaming conditions. The results provide insights into the degradation mechanisms, potential synergistic effects between components, and the overall efficiency of the flame-retardant systems studied.

### 3.1. Dispersion

**Figure 1:**
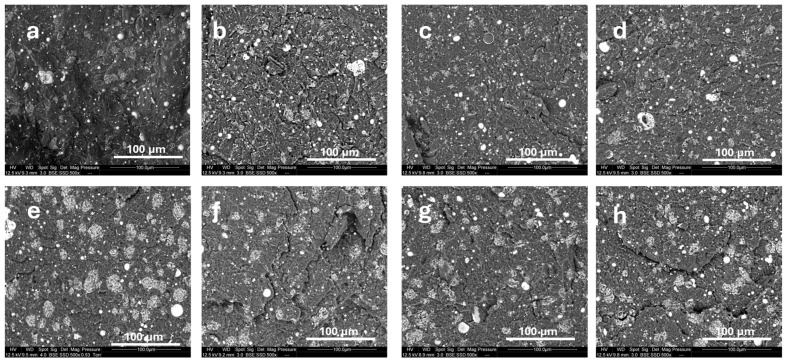
SEM-BSE images of polyamide composites containing 12.5 wt% flame retardant (melamine polyphosphate: (**a**–**d**); aluminum diethyl phosphinate: (**e**–**h**)) and 12.5 wt% fly ash. The fly ash was untreated (**a**,**e**) or surface-treated with amino-silane (**b**,**f**), glycidoxy-silane (**c**,**g**), or titanate (**d**,**h**).

The dispersion of fly ash particles in the polyamide matrix was examined using scanning electron microscopy (SEM), as shown in Figure 1. Images a–d correspond to composites containing melamine polyphosphate (MPP) and surface-treated fly ash, while images e–h show the morphology of composites with aluminum diethyl phosphinate (OP) and untreated or modified fly ash.

In the MPP-based formulations (b–d), the micrographs reveal noticeable differences in the distribution of the inorganic phase depending on the surface treatment. In image b, corresponding to amino-silane treatment, the particles appear relatively well distributed, with limited visible agglomeration. Image c, corresponding to glycidoxy-silane treatment, shows a similar morphology, although some larger aggregates remain visible. In image d, corresponding to titanate-treated fly ash, the inorganic domains appear more finely distributed within the matrix, with fewer large agglomerates, suggesting a more homogeneous filler distribution.

In the OP-based formulations (e–h), the differences are even more pronounced. In image e, where untreated fly ash was used, large agglomerates are clearly visible, indicating a less homogeneous distribution of the mineral phase within the matrix. After amino-silane treatment (f), the morphology becomes more uniform, with smaller and more dispersed inorganic domains. Glycidoxy-silane treatment (g) also leads to a more homogeneous distribution, although some localized clustering can still be observed. Among the OP-based formulations, titanate-treated fly ash (h) exhibits the finest apparent distribution and the lowest extent of visible aggregation.

Overall, these observations indicate that surface treatment modifies the morphology of the inorganic phase in PA6 and tends to improve its distribution, especially in the case of titanate treatment. This evolution is consistent with the fire behavior results obtained by cone calorimetry, indicating that the distribution of the mineral phase likely plays a role in the condensed-phase response of the composites during combustion.

### 3.2. Thermal Degradation

**Figure 2:**
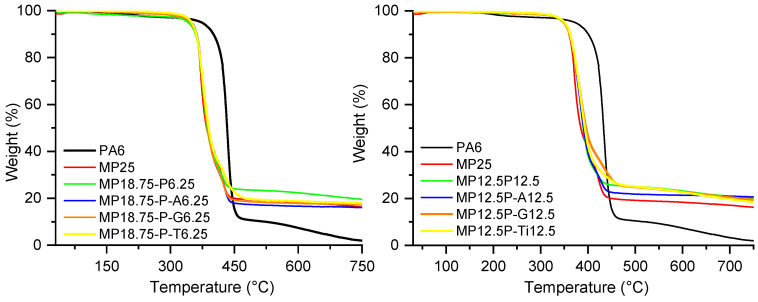
TGA results of PA6 formulations containing melamine polyphosphate.

Thermogravimetric analysis (TGA) was carried out under nitrogen to evaluate the thermal stability of the various PA6 formulations. Key parameters extracted from the TGA curves include the temperatures at 5%, 20%, and 50% weight loss (T_5_%, T_20_%, T_50_%), as well as the residual mass at 750 °C. Results are summarized in Table 1 and Table 2. The unfilled PA6 exhibited an onset of degradation at 373 °C and left only 1.7% residue at 750 °C. In contrast, the formulation containing 25 wt% melamine polyphosphate (MP25) showed (Table 1 and Figure 2) an earlier degradation onset (T_5_% = 349 °C), as expected from the decomposition of MPP, but resulted in a significantly higher char yield of 16%. A similar trend was observed in the MPP-based formulations combined with 6.25 or 12.5 wt% of untreated or modified fly ash. These blends generally exhibited T_5_% values in the range of 342–351 °C and produced between 16 and 21% residue, indicating an effective contribution of MPP to condensed-phase char formation. Notably, the combination MP12.5P-A12.5 led to the highest residue (21%), suggesting that the aminosilane surface treatment of the filler may enhance char stabilization.

The dTG curves provided in the Supporting Information confirmed that the MP-containing filled formulations degraded in two main steps, with peak temperatures between 373 and 386 °C and 412–437 °C, whereas neat PA6 showed a single maximum at 434 °C.

**Figure 3:**
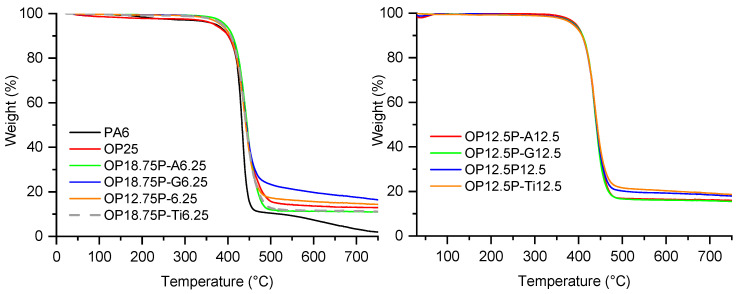
TGA results of PA6 formulations containing aluminum diethyl phosphinate.

Formulations containing 25 wt% of aluminum phosphinate (OP25, Figure 3 and Table 2) were thermally more stable than their MPP counterparts, with T_50_% reaching up to 442 °C and a residue of 13%. When OP was combined with untreated or surface-modified fly ash, the degradation onset was shifted to higher temperatures, particularly in the presence of APTES or GPTES-treated fillers. For example, OP18.75P-A6.25 exhibited a T_5_% of 394 °C and a T_50_% of 445 °C, indicating enhanced thermal stability, although the final residue was slightly reduced (11%) compared to the unmodified filler (14–16%). The formulations with 12.5/12.5 OP/P combinations showed similar trends, with T_5_% values between 387 and 394 °C and residues ranging from 15 to 18%.

The dTG curves provided in the Supporting Information showed that all OP-containing formulations degraded in a single main step, with peak temperatures between 436 and 446 °C (Table 2), compared with 434 °C for neat PA6.

Overall, the use of OP systematically improved thermal stability compared to MPP, while MPP-based systems generated higher residual masses due to their inherent char-forming mechanisms. The presence of mineral fillers, particularly when surface-treated, further influenced both decomposition temperatures and residue formation, underlining their synergistic role in enhancing the condensed-phase performance of the flame retardant systems.

### 3.3. Fire Behavior Measured by Cone Calorimeter and UL94

**Figure 4:**
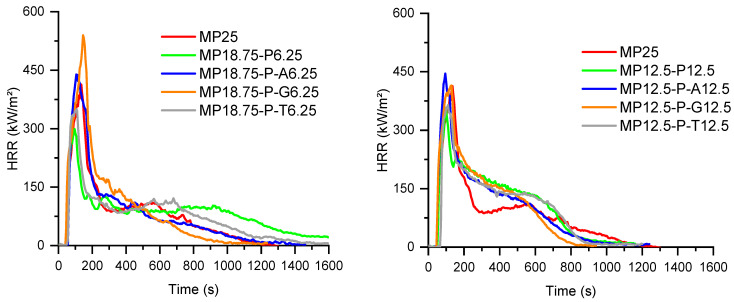
HRR curves of PA6 formulations containing melamine polyphosphate and fly ash.

**Figure 5:**
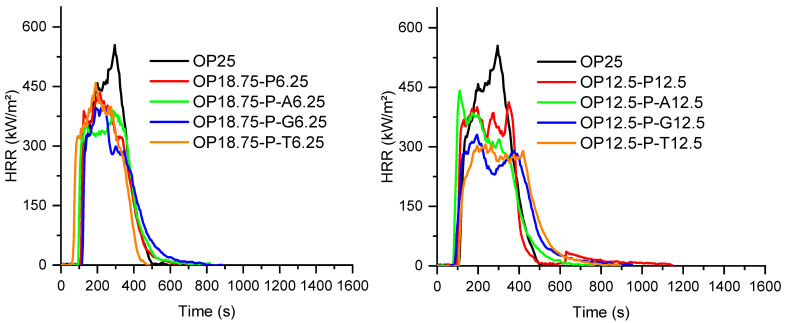
HRR curves of PA6 formulations containing aluminum diethyl phosphinate and fly ash.

The fire behavior of the PA6 formulations was assessed under forced-flaming conditions using a cone calorimeter (50 kW/m^2^, ISO 5660-1). The main fire parameters—time to ignition (TTI), peak heat release rate (pHRR), total heat release (THR), mean average heat release rate (MAHRE), and char residue—were combined with UL-94 vertical burning results to obtain a comprehensive view of both combustion dynamics and flame self-extinguishing capability. These parameters are particularly relevant for assessing the flame retardant efficiency in real fire scenarios, as they reflect both ignition resistance and fire growth potential. The results are summarized in Table 3 and Table 4 and Figure 4 and Figure 5 and provide insights into the influence of the flame retardant type, filler content, and surface treatment on the combustion performance of PA6.

As expected, neat PA6 exhibited poor fire performance, with a short time to ignition (TTI = 79 s), an extremely high peak heat release rate (pHRR ≈ 1350 kW/m^2^), and a total heat release (THR) of 150 MJ/m^2^, consistent with its well-known flammability. The addition of 25 wt% melamine polyphosphate (MP25 formulation, Figure 4) significantly reduced the pHRR to 389 kW/m^2^ and lowered the THR to 118 MJ/m^2^. These improvements are attributed to the endothermal decomposition of MPP, which releases inert gases and promotes char formation, resulting in a residue of 12%. However, the TTI of MP25 decreased to 51 s. This behavior may be related to the relatively low decomposition temperature of MPP, which leads to the early release of ammonia, melamine-derived species, and phosphorus-containing compounds [10,14]. These products are likely to modify the initial degradation pathway of PA6 under heat flux by promoting condensed-phase reactions such as dehydration and char formation, while gaseous products contribute to the dilution of combustible volatiles. This interpretation is supported by the TGA results, which show a lower onset degradation temperature when MPP is incorporated.

In contrast, the OP25 formulation showed a longer TTI (100 s) and lower pHRR (587 kW/m^2^) than neat PA6, though not as low as MP25. Despite this, its MAHRE remained relatively high (306 kW/m^2^), and the residue was limited to 3.5%, suggesting that aluminum phosphinate acts more strongly in the gas phase, with limited char formation in this specific formulation.

When MPP or OP were combined with fly ash (SP), either untreated or surface-treated, further improvements were observed.

In MPP-based systems, the addition of 6.25 or 12.5 wt% SP leads to mixed results (Figure 4). On the whole, except for MP12.5P-T12.5, the TTI values are reduced, whereas only some compositions with fly ash help reduce pHRR, THR and MAHRE. For both SP loadings, only the compositions without surface treatment and with the use of titanate as a surface agent lead to a significant improvement of the fire reaction parameters in comparison with MP used alone, showing a synergistic effect between the flame retardant and the mineral filler. In all cases, the presence of SP allows the final quantity of residue to be increased.

Conversely, for all OP-based formulations, the combination of OP1230 with SP or treated SP always led to an improvement in fire behavior, showing a synergistic effect between OP and SP (Figure 5). At 6.25 wt% SP, titanate-modified SP again significantly improved the fire performance, showing a reduced pHRR (361 kW/m^2^), MAHRE (175 kW/m^2^) and an increased residue (15.7%), despite a short TTI (57 s). At 12.5 wt% SP, only surface treatments with titanate and GPTES lead to equivalent or better performance than those of the composition with untreated SP. For most OP compositions with SP, the amount of final residue is higher than the sum of the initial amount of SP and the amount of residue for OP used alone. This shows that the presence of SP promotes the cohesion of a condensed phase during the combustion of the material.

The Fire Growth Rate Index (FIGRA, determined from cone calorimeter data as $\mathrm{pHRR}/t_{\mathrm{pHRR}}$, where $t_{\mathrm{pHRR}}$ is the time required to reach the peak heat release rate) was used as an additional parameter to assess the fire hazard of the different formulations. Since lower FIGRA values indicate slower fire development, this parameter provides useful complementary information to pHRR and THR. Neat PA6 displayed the highest FIGRA value (5.1), confirming its high fire hazard. All flame-retarded formulations showed lower FIGRA values, indicating a significant reduction in fire growth.

In the MPP-based series, MP25 showed a FIGRA of 3.34, while partial replacement of MPP by untreated fly ash slightly reduced this value to 3.13 and 3.00 for MP18.75P6.25 and MP12.5P12.5, respectively. The effect of surface treatment depended strongly on the modifier. Amino-silane-treated fly ash led to higher FIGRA values, whereas glycidoxy-silane gave intermediate results. Titanate-treated fly ash was the most effective in reducing fire growth, with FIGRA values of 2.41 for MP18.75P-T6.25 and 1.29 for MP12.5P-T12.5.

In the OP-based series, OP25 already exhibited a low FIGRA value (1.88). Untreated fly ash and glycidoxy-silane-treated fly ash maintained or further improved this behavior, with the lowest FIGRA value obtained for OP12.5P12.5 (1.18). By contrast, titanate-modified fly ash significantly increased FIGRA in the OP formulations, reaching 3.33 and 3.31 for OP18.75P-T6.25 and OP12.5P-T12.5, respectively. Therefore, the FIGRA results show that the influence of fly ash surface treatment depends strongly on the phosphorus flame retardant considered: titanate treatment is beneficial in the MPP system, whereas it is unfavorable in the OP system.

Overall, cone calorimeter results indicate that both MPP and OP are effective in reducing the flammability of PA6, but their efficiency can be significantly improved by incorporating fly ash, above all for OP. Surface treatments, particularly with titanate, further enhance fire retardancy by promoting better dispersion, stronger filler–matrix interaction, and more stable char formation. The best-performing systems combine condensed-phase and gas-phase mechanisms, resulting in reduced fire growth rate, heat release, and increased residue after combustion.

Regarding the ability to self-extinguish for the various compositions, UL-94 testing highlights a strong contrast between the two phosphorus-based flame retardants. All MPP-containing formulations (Table 3), with or without fly ash or surface-treated fly ash, achieve only V-2, indicating that although MPP enhances char formation, it does not sufficiently limit dripping or promote rapid flame extinction in vertical burning. Conversely, all OP-based formulations consistently reach the V-0 rating, independent of fly ash content or surface modification (Table 4). This superior performance results from the gas-phase flame-quenching activity of aluminum diethyl phosphinate, which efficiently suppresses flaming and prevents dripping. Although fly ash can reinforce condensed-phase stability, even in the case of OP, with a significant increase in the amount of residue, the UL-94 classification remains dominated by the intrinsic mechanism of each flame-retardant system.

## 4. Discussion

The cone calorimeter data confirms the effectiveness of both melamine polyphosphate (MPP) and aluminum phosphinate (OP1230) as flame retardants for PA6, yet they also reveal distinct differences in their modes of action and interactions with mineral fillers.

While MPP-containing formulations consistently show higher char yields (up to 21%), they exhibit shorter ignition times, indicating that the decomposition of MPP occurs at lower temperatures, releasing non-flammable gases (e.g., ammonia, melamine derivatives) that dilute the flammable volatiles [13,33]. This gas-phase dilution, combined with the promotion of char formation in the condensed phase, contributes to the significant reduction in the peak heat release rate (pHRR). However, the early decomposition also limits their ability to self-extinguish. These findings are supported by phosphorus balance measurements (Table 5), which correspond to the ratio between the remaining phosphorus in the residue and the initial amount. This shows that systems with higher MPP content retain more phosphorus in the condensed phase. Moreover, the presence of fly ash tends to increase this amount. This can be ascribed to the formation of new species containing both silicon and phosphorus. For instance, MP25 retains 55.8% of the initial phosphorus, while the binary system MP18.75/P6.25 shows a significantly higher retention of 74.7%. It also has to be noticed that even for OP, which acts predominantly in the gaseous phase, a significant amount of phosphorus remains trapped in the condensed phase.

The role of fly ash, whether untreated or surface-modified, appears to be predominantly synergistic. Its incorporation enhances the condensed-phase barrier effect by stabilizing the char layer and possibly contributing to the formation of ceramic-like structures upon combustion [28,29]. Visual inspection of the cone calorimeter residues indicated generally compact and non-expanded chars, with relatively limited surface cracking, suggesting the formation of a relatively cohesive protective layer. This is particularly evident in binary systems where the combination of fly ash with either flame retardant leads to simultaneous reductions in pHRR and increases in residue. These treatments are likely to improve the dispersion of the filler and the interfacial adhesion with the polymer matrix, thus enhancing the structural integrity of the protective char.

In OP-based formulations, where flame retardancy is mainly governed by gas-phase mechanisms, the presence of surface-modified fly ash still provides a measurable benefit through char reinforcement. For instance, titanate-modified SP in the OP-based formulation (OP18.75/P-Ti12.5) led to both a reduced pHRR and an increased residue (15.7%), although its phosphorus balance remained relatively low (26.7%), consistent with the predominant gas-phase action of OP.

In contrast, systems containing melamine polyphosphate (MPP), which primarily acts in the condensed phase, exhibit a markedly different behavior. The formulations MP12.5/P-A12.5, MP12.5/P-G12.5, and MP12.5/P-Ti12.5 all show complete phosphorus retention (100%, Table 5), indicating a highly efficient trapping of phosphorus-containing species within the condensed phase. This suggests a strong interaction between the flame retardant and the surface-modified fly ash, promoting the formation of thermally stable char structures. Consequently, these systems display a pronounced suppression of heat release together with increased char yields (>15%).

**Figure 6:**
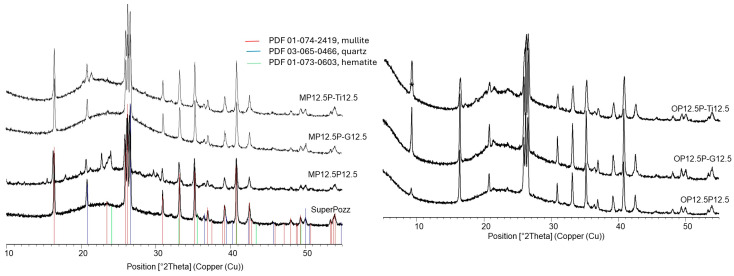
XRD diffractograms of fly ash and selected cone calorimeter residues.

XRD analyses of selected cone calorimeter residues were carried out to investigate possible interactions between fly ash and the phosphorus-based flame retardants in the condensed phase (Figure 6). The main diffraction peaks observed in the residues can still be assigned to the crystalline phases initially present in the fly ash, mainly quartz and mullite, indicating that the mineral framework of the filler is preserved during combustion. In addition, differences in peak positions and relative intensities between the formulations indicate that the final residue is not merely a physical mixture of the initial inorganic constituents but results from transformations occurring during combustion.

These observations support the idea that fly ash participates in the stabilization of the condensed phase and interacts with phosphorus-containing species released by the flame retardants. In agreement with the phosphorus balance measurements, this may promote the formation of phosphorus-containing mineral-rich structures within the residue. Nevertheless, since no clearly identifiable new crystalline phosphate phase could be unequivocally assigned, the phosphorus-rich protective structures formed during combustion are likely to be at least partly amorphous or poorly organized. This is consistent with the barrier effect inferred from the lower pHRR and higher residue contents obtained in cone calorimetry.

**Figure 7:**
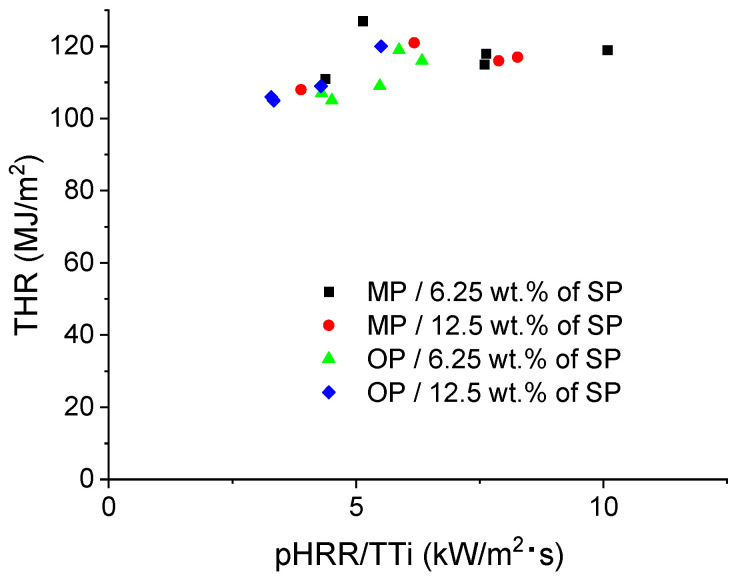
Petrella plot [34]: THR versus PHRR/TTi to assess fire behavior.

To further assess the overall fire hazard of the different formulations, a Petrella-type plot [34] was constructed by plotting THR as a function of pHRR/TTI (Figure 7). This representation combines the total heat released during combustion with the rate of fire growth after ignition. In this context, lower pHRR/TTI values indicate a lower tendency for rapid fire development and thus a lower relative flashover propensity, while lower THR values reflect a reduced overall fire load. The most favorable formulations are therefore located in the lower-left region of the plot.

The plot highlights clear differences between the two flame-retardant systems. In the MPP-based series, titanate-treated fly ash leads to the most favorable shift, especially for MP18.75/P-Ti6.25 and MP12.5/P-Ti12.5, which combine lower pHRR/TTI values with reduced THR. This indicates a better balance between ignition behavior and heat release reduction. By contrast, amino- and glycidoxy-silane treatments remain associated with higher pHRR/TTI values, reflecting faster fire growth despite reduced heat release compared with neat PA6.

In the OP-based series, several formulations are located in a more favorable region of the plot, confirming the lower overall fire hazard of these systems. In particular, OP12.5/P12.5 and OP12.5/P-G12.5 combine low THR with low pHRR/TTI, indicating reduced fire growth and lower relative flashover propensity. In contrast, titanate-modified fly ash appears less beneficial in the OP system, as it shifts the formulations toward higher pHRR/TTI values. Overall, this analysis confirms that the influence of fly ash surface treatment depends strongly on the phosphorus flame retardant considered.

These observations support the hypothesis that surface-treated mineral fillers play a crucial role by ensuring better integration of the filler into the matrix and promoting more cohesive and thermally stable char structures, as evidenced by both enhanced residue formation and improved phosphorus retention.

## 5. Conclusions

The present study demonstrates that the surface modification of fly ash particles plays a crucial role in enhancing the fire performance of polyamide 6 (PA6) composites containing phosphorus- and nitrogen-based flame retardants. The incorporation of melamine polyphosphate (MPP) and aluminum diethyl phosphinate (OP1230), either alone or in combination with untreated and surface-treated fly ash, significantly influenced both the thermal degradation behavior and the combustion performance of the resulting materials.

Thermogravimetric analyses revealed that MPP-based systems favored char formation, while OP1230-based formulations exhibited greater thermal stability, particularly at higher temperatures. The addition of fly ash, and more notably of surface-modified fly ash, led to an increase in the residue content and a shift in degradation temperatures to higher values. This improvement was attributed to enhanced filler–matrix interactions and the stabilization of the condensed phase through the formation of ceramic-like structures. Titanate treatment proved particularly effective, promoting superior filler dispersion and stronger interfacial adhesion within the PA6 matrix.

Cone calorimeter results confirmed the beneficial role of surface modification. All formulations containing treated fly ash exhibited a marked reduction in the peak heat release rate (pHRR) and total heat release (THR) compared with neat PA6, with reductions up to 75% depending on the formulation. The presence of titanate- and silane-treated fillers improved both the cohesion and integrity of the protective char layer, leading to lower heat and mass transfer during combustion.

Elemental analyses of the combustion residues confirmed synergistic action between the phosphorus FRs and SP, showing nearly complete phosphorus retention in the composites containing both flame retardants and surface-treated fly ash. This high phosphorus retention indicates the formation of stable inorganic structures such as aluminophosphates or silicate-based networks, which reinforce the protective barrier formed during burning.

Overall, the results highlight that surface-modified fly ash acts as an efficient synergistic additive in flame-retardant PA6 systems, combining improved fire behavior with enhanced filler dispersion and interfacial adhesion. Beyond the technical benefits, the use of fly ash also represents a sustainable approach to material design, promoting the valorization of industrial by-products and reducing reliance on virgin mineral fillers while also contributing to the conservation of phosphorus resources, which are crucial and limited elements. These findings open promising perspectives for the development of eco-efficient, high-performance polyamide materials combining fire safety, mechanical performance, and environmental responsibility.

## Figures and Tables

**Table 1:** Thermal degradation data for the PA6/melamine polyphosphate formulations.

Formulation	T_5%_ (°C)	T_20%_ (°C)	T_50%_ (°C)	T_pic1_ (°C)	T_pic2_ (°C)	Residue at 750 °C (%)
PA6	373	419	433	434	-	1.7
MP25	349	368	385	373	425	16
MP18.75P6.25	342	369	386	373	412	19
MP18.75P-A6.25	350	371	388	374	428	16
MP18.75P-G6.25	347	369	387	375	428	17
MP18.75P-T6.25	349	370	390	374	427	18
MP12.5-P12.5	348	372	390	381	415	20
MP12.5P-A12.5	350	372	391	374	428	21
MP12.5P-G12.5	349	372	396	385	435	19
MP12.5P-T12.5	351	372	393	386	437	18

**Table 2:** Thermal degradation data for the PA6/aluminum diethyl phosphinate formulations.

Formulation	T_5%_ (°C)	T_20%_ (°C)	T_50%_ (°C)	T_pic1_ (°C)	Residue at 750 °C (%)
PA6	373	419	433	434	1.7
OP25	369	420	442	444	13
OP18.75P6.25	384	421	442	445	14
OP18.75P-A6.25	394	425	445	444	11
OP18.75P-G6.25	385	422	443	446	16
OP18.75P-T6.25	388	422	443	445	11
OP12.5P12.5	388	422	441	436	18
OP12.5P-A12.5	394	422	441	439	16
OP12.5P-G12.5	391	423	440	438	15
OP12.5P-T12.5	387	422	442	439	18

**Table 3:** Cone calorimeter and UL94 results of PA6 formulations containing melamine polyphosphate and fly ash.

Formulation	TTI (s)	pHRR (kW/m^2^)	THR (MJ/m^2^)	MAHRE (kW/m^2^)	FIGRA (kW/(m^2^·s)	Residue (%)	UL94
PA6	79 ± 2	1352 ± 180	150 ± 5	406 ± 30	5.1	-	NR
MP25	51 ± 3	389	118 ± 4	211 ± 28	3.34	12 ± 0.2	V-2
MP18.75P6.25	59 ± 2	303	127 ± 3	239 ± 25	3.13	14 ± 0.1	V-2
MP18.75P-A6.25	57.5 ± 0.7	433 ± 8	115 ± 3	238 ± 5	4.18	13.5 ± 0.5	V-2
MP18.75P-G6.25	50.5 ± 6	504 ± 49	119.5 ± 2	258 ± 25	3.34	14.4 ± 0.8	V-2
MP18.75P-T6.25	82 ± 15	359 ± 30	111 ± 1.5	281 ± 8	2.41	18.5 ± 0.3	V-2
MP12.5P12.5	57 ± 2	352 ± 20	121 ± 1	183 ± 6	3	20 ± 0.3	V-2
MP12.5P-A12.5	53 ± 7	438 ± 10	117.5 ± 0.7	209 ± 18	4.61	21 ± 0.6	V-2
MP12.5P-G12.5	51 ± 3	402 ± 18	116.5 ± 0.7	217 ± 20	3.18	18.5 ± 0.1	V-2
MP12.5P-T12.5	98 ± 1	381 ± 107	108 ± 2	234 ± 47	1.29	15.7 ± 0.8	V-2

**Table 4:** Cone calorimeter and UL94 results of PA6 formulations containing aluminum diethyl phosphinate and fly ash.

Formulation	TTI (s)	pHRR (kW/m^2^)	THR (MJ/m^2^)	MAHRE (kW/m^2^)	FIGRA (kW/(m^2^·s))	Residue (%)	UL94
PA6	79 ± 1	1352 ± 40	150 ± 2	406 ± 32	5.1	-	NR
OP25	100 ± 9	587 ± 45	119 ± 1	306 ± 29	1.88	3.5 ± 0.1	V-0
OP18.75P6.25	105 ± 6	452 ± 26	107 ± 0.8	268 ± 25	2.01	10.7 ± 0.5	V-0
OP18.75P-A6.25	77 ± 15	422 ± 42	109.5 ± 0.8	270 ± 30	1.36	10.6 ± 0.3	V-0
OP18.75P-G6.25	94 ± 25	424 ± 35	105.5 ± 3	253 ± 50	1.65	10.3 ± 0.1	V-0
OP18.75P-T6.25	57 ± 1	361 ± 16	116 ± 1	175 ± 9	3.33	15.7 ± 0.5	V-0
OP12.5P12.5	108 ± 13	355 ± 79	106.5 ± 2	221 ± 58	1.18	17.5 ± 0.5	V-0
OP12.5P-A12.5	89 ± 10	382 ± 45	109.5 ± 2	231 ± 34	4	15.4 ± 1.8	V-0
OP12.5P-G12.5	92 ± 2	307 ± 31	105 ± 0.8	201 ± 6	1.69	16 ± 0.4	V-0
OP12.5P-T12.5	67 ± 1	369 ± 6	120 ± 1.5	179 ± 1	3.31	17.6 ± 0.2	V-0

**Table 5:** EDX results of cone calorimeter residues of selected formulations.

Sample	Phosphorus Balance
MP25	55.8
MP18.75/P6.25	74.7
MP12.5/P12.5	94
MP12.5/P-A12.5	100
MP12.5/P-G12.5	100
MP12.5/P-Ti12.5	100
OP12.5/P12.5	29.1
OP12.5/P-A12.5	23.9
OP12.5/P-G12.5	27.6
OP18.75/P-Ti12.5	26.7

## Data Availability

The raw data supporting the conclusions of this article will be made available by the authors on request.

## Supplementary Materials

The following supporting information can be downloaded at: https://www.mdpi.com/article/10.3390/polym18080970/s1, Figure S1: TGA results of SuperPozz (SP), melamine polyphosphate (MP200) and aluminum diethyl phosphinate (OP1230); Figure S2: dTG results of PA6 formulations containing aluminum melamine polyphosphate; Figure S3: dTG results of PA6 formulations containing aluminum diethyl phosphinate.
