# Supplementary material for: Influence of Surface Modification of Fly Ashes on the Fire Behavior of Polyamide 6
Source: Polymers (Basel). 2026 Apr 16;18(8):970. doi: 10.3390/polym18080970 (PMC13120369; doi:10.3390/polym18080970)
Supplement: Supplementary file 1 [file polymers-18-00970-s001.zip › polymers-4187631-supplementary.pdf]

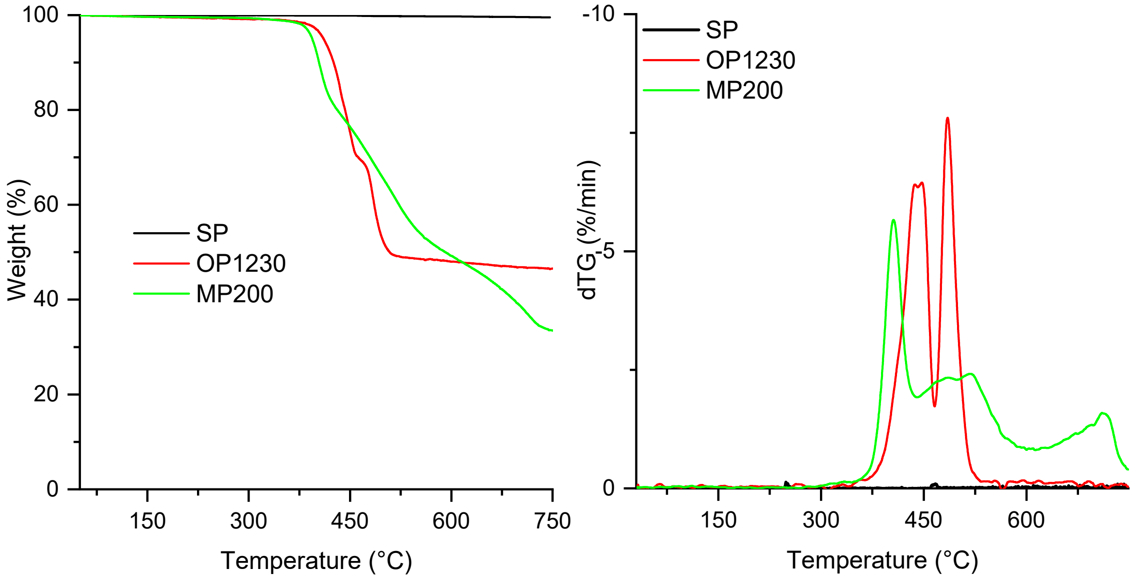

Figure S1. TGA results of SuperPozz (SP), melamine polyphosphate (MP200) and aluminum diethyl phosphinate (OP1230).

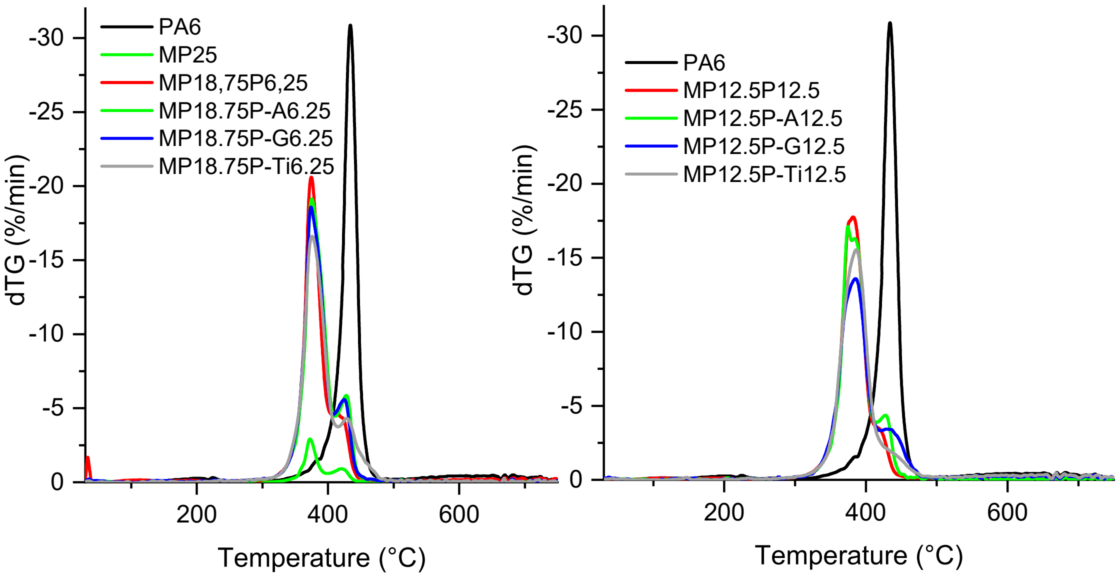

Figure S2. dTG results of PA6 formulations containing aluminum melamine polyphosphate.

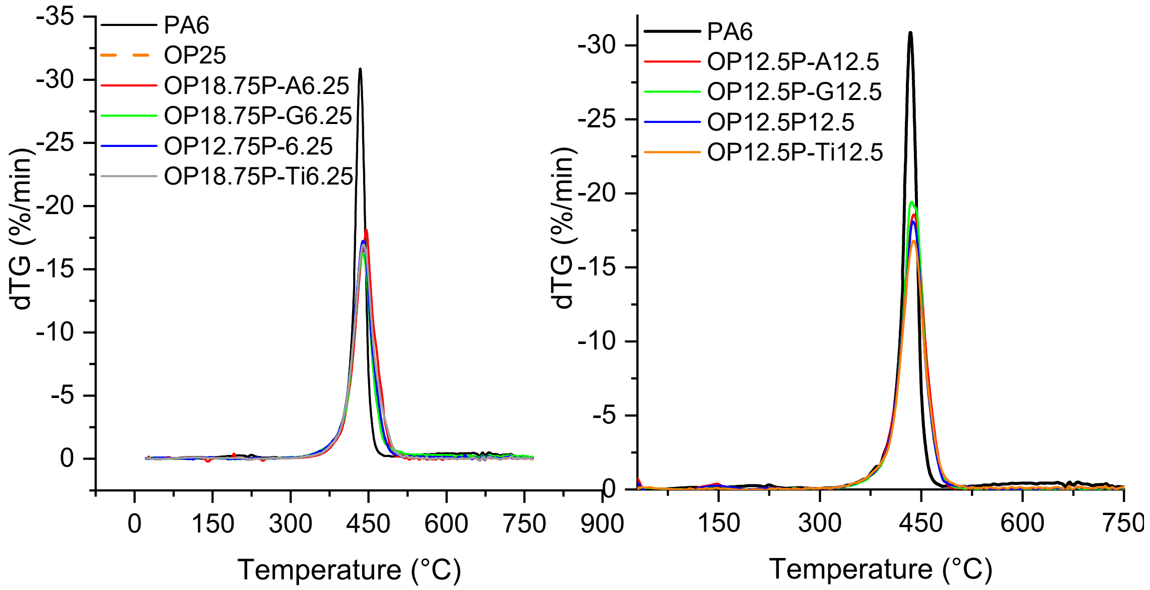

Figure S3. dTG results of PA6 formulations containing aluminum diethyl phosphinate.
